# Multiple Intracranial Rosai-Dorfman Disease: A Case Report

**DOI:** 10.7759/cureus.5292

**Published:** 2019-07-31

**Authors:** Evan M Krueger, Henry G Brown, Keith Schaible

**Affiliations:** 1 Neurosurgery, Advocate Health Care, Downers Grove, USA; 2 Pathology, Advocate Christ Medical Center, Oak Lawn, USA; 3 Neurosurgery, Advocate Christ Medical Center, Oak Lawn, USA

**Keywords:** rosai-dorfmann disease, intracranial, extra-axial

## Abstract

Rosai-Dorfman disease is an uncommon lymphoproliferative disorder, and multiple intracranial involvement in disseminated disease is exceedingly rare. We present a case of a 52 year-old female who presented with intractable headaches and a history of Rosai-Dorfman disease unresponsive to chemo- and radiation therapies. She was found to have new multiple intracranial masses that were treated with surgical excision. Pathology confirmed a diagnosis of intracranial Rosai-Dorfman. The disease presentation, radiographic appearance, histology, treatment, and prognosis are briefly reviewed.

## Introduction

Rosai-Dorfman disease (RDD) is an idiopathic benign lymphoproliferative disorder. The mean age of diagnosis is 39 years old, there is a slight female preponderance (1.8:1), and the estimated yearly incidence in the United States is 100 cases [1,2]. RDD is characterized by emperipolesis of lesional histiocytes, typically in painless enlarged cervical lymph nodes [3]. We present a case of disseminated RDD refractory to chemo- and radiation therapies, that presented with multiple intracranial manifestations.

## Case presentation

A 52 year-old female presented with a two-day history of insidious, progressive, and intractable right occipital-temporal headache. She had a past medical history of RDD diagnosed five years prior to presentation. At that time she underwent two rounds of chemotherapy with adjunct steroids over a two year time period, with persistent enlarged, painless cervical lymphadenopathy. Two years prior to presentation, she experienced bilateral parotid gland enlargement. She then underwent 500 cGy in 15 fractions to the left parotid gland, and 1200 cGy to the right parotid gland, followed one year later by a boost of 1400 cGy in seven fractions to the right parotid gland, that did not shrink the size of these masses. Her last cranial imaging was done two years prior to presentation.

**Figure 1:**
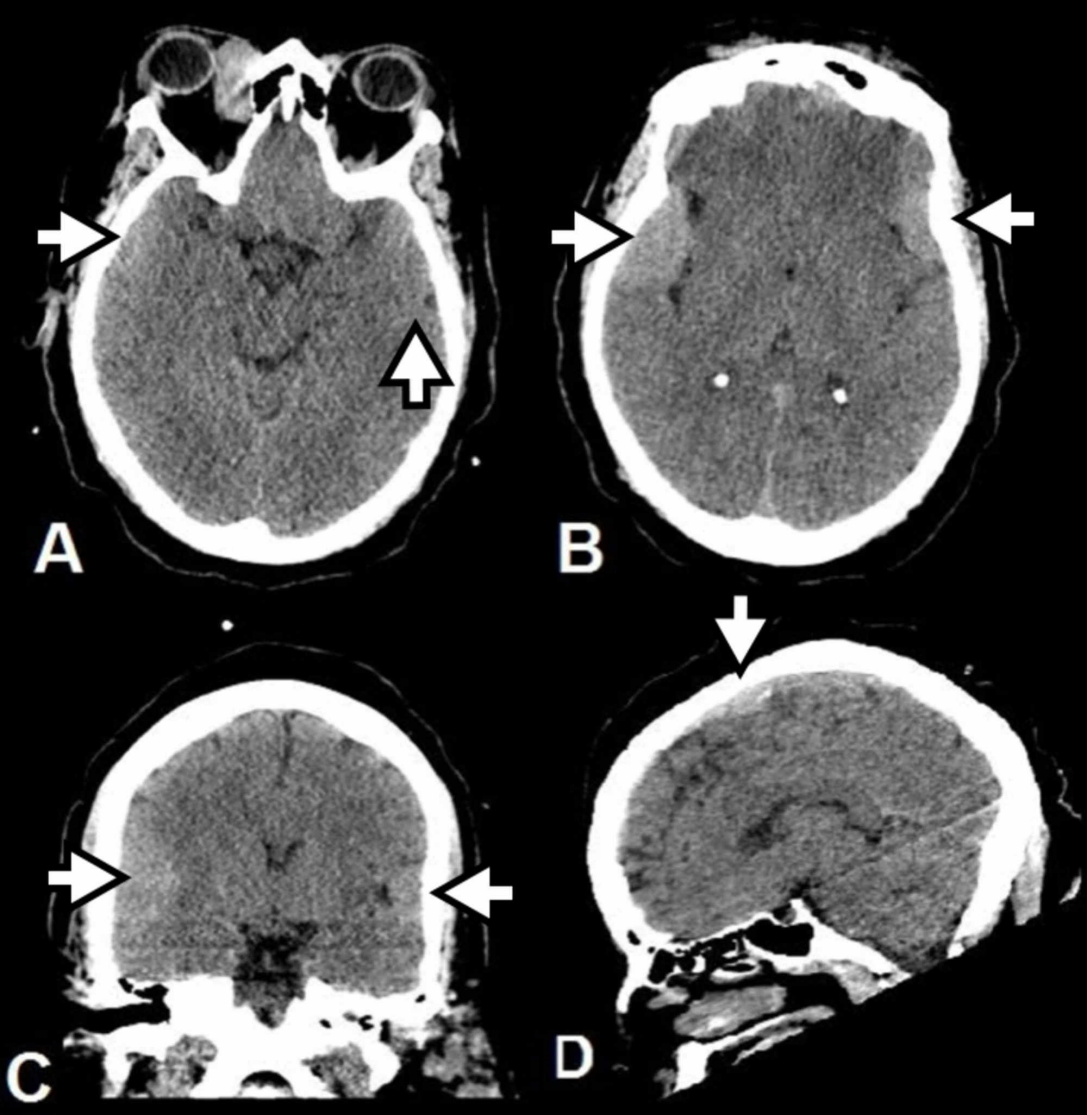
Screening Computed Tomography Head Scans Axial (A&B), coronal (C), and sagittal (D) slices show multiple extra-axial hyperdense masses with effacement of the adjacent sulci and gyri. The largest in the right frontal-temporal region measures 4.0 centimeters anterior-posterior and 3.7 centimeters cranial-caudal.

**Figure 2:**
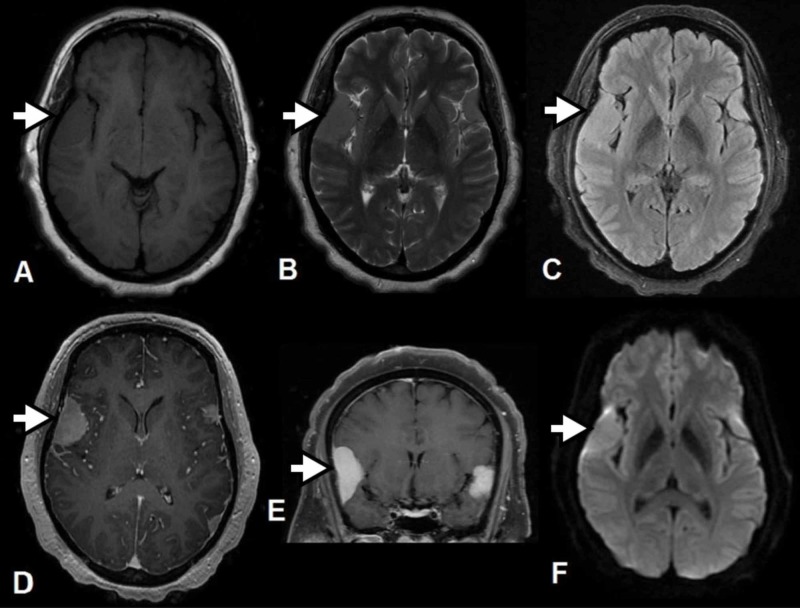
Magnetic Resonance Imaging With and Without Contrast Images Axial T1 (A), axial T2 (B), axial FLAIR (C), axial and coronal T1 post-contrast (D&E), and axial DWI (F) sequences show dural based extra-axial supratentorial lesions along the bilateral paramidline frontal and right parietal lobes at the vertex, left frontal lobe near the vertex, bilateral posterior parietal lobes, and right greater than left frontal-temporal lobes. These lesions were homogeneously enhancing, partially diffusion restricting, and without vasogenic edema in the adjacent parenchyma.

**Figure 3:**
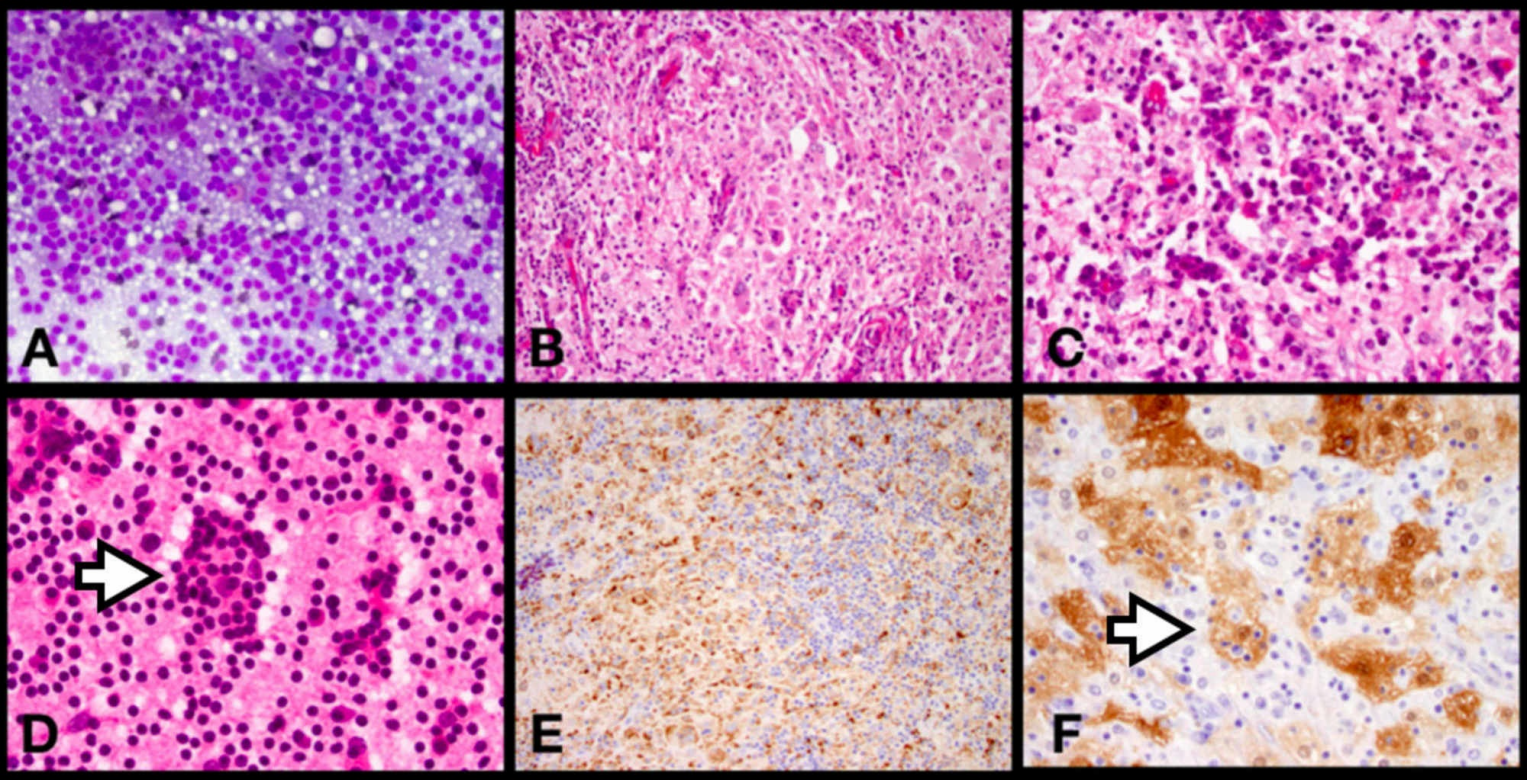
Right Frontal-Temporal Extra-Axial Mass Histopathology Diff quik (A), low power H&E photomicrograph (B) and medium power H&E photomicrograph showing a mixed population of macrophages and lymphocytes (C), H&E smear prep highlighting a macrophage with lymphocytic emperipolesis (D), CD68+ macrophages with negative lymphocytes and plasma cells (E), and S100+ macrophages with emperipolesis (F).

Updated computed tomography (CT) (Figure 1) and magnetic resonance imaging (MRI) (Figure 2) head scans were obtained on presentation, which showed a new diagnosis of multiple extra-axial lesions; the largest of which was in the right frontal-temporal region. Given her intractable pain, uncertain etiology of these new extra-axial masses, and poor response to previous treatment modalities for RDD, she underwent a right frontotemporal craniotomy for resection of her largest lesion only. Gross total resection was achieved. Histopathology confirmed intracranial involvement of RDD (Figure 3).

**Figure 4:**
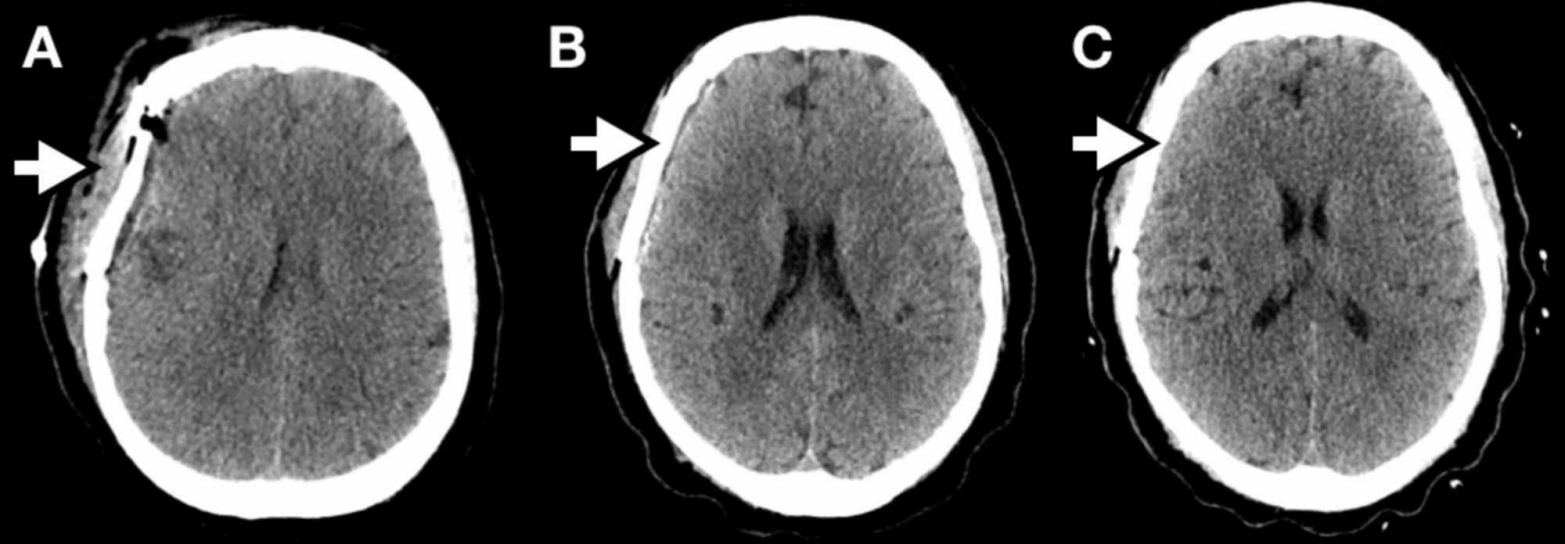
Post-operative computed tomography head images Surveillance images obtained one day (A), one month (B), and three months (C) post-operatively show gross total resection of the right frontal-temporal lesion. There are multiple known, persistence extra-axial lesions.

Post-operative imaging showed gross total resection of the right frontal lesion (Figure 4). Her post-operative course was remarkable only for a seizure controlled with a single anti-epileptic agent, and she was discharged home. Follow up images at one and three months post-operatively were negative for recurrence (Figure 4). She is being followed regularly by hematology, and there are no plans for additional chemo- or radiation therapies.

## Discussion

Here we present an atypical presentation of an uncommon disease. Central nervous system (CNS) involvement in RDD is rare, with only 210 total cases reported [1]. CNS RDD usually occurs without extracranial lymphadenopathy: only 36 cases of disseminated disease with CNS involvement have been reported [1]. Furthermore, only 18 cases of multiple intracranial RDD have been described [4].

RDD classically presents as massively enlarged, painless cervical lymphadenopathy. Common extra-nodal sites include skin, upper respiratory tract, and bone [3]. Constitutional symptoms are typically present [3]. The most common intracranial sites include the cranial base, parasellar and suprasellar regions, ventricles, and convexity [4]. Accompanying neurologic symptoms vary by mass location.

Radiographically, RDD presents as a single or multiple dural based masses, and can easily be mistaken for a meningioma. On CT, it is hyperdense, lobulated, with heterogeneous enhancement [5]. Hyperostosis, bone erosion, and calcification are typically absent but can be common in meningiomas. On MRI T1 weighted imaging, it is isointense with distinct, strong, heterogenous enhancement and absent dural tail; and on MRI T2 weighted imaging, it is iso- to hyperintense [5]. RDD displays a lower apparent diffusion coefficient than meningioma but higher than lymphoma [5]. Additionally, magnetic resonance spectroscopy shows a choline peak of 140 ppm, unlike a meningioma which typically shows an alanine peak near 48 ppm [5]. Differentials to consider include meningioma, lymphoma, dural based metastasis, Langerhan’s histiocytosis, Wegener’s granulomatosis, sarcoidosis, and pseudotumor.

The definitive diagnosis of RDD is dependent on histology. Typical findings include large well-defined histiocytes with phagocytic vacuoles of intact lymphocytes and erythrocytes found within their cytoplasm (emperipolesis). A dense, mixed, chronic cellular inflammatory infiltrate is also present. Positive staining for S100 and CD68 confirms the presence of histiocytes and can help to exclude granulomatous and infectious diseases. To exclude RDD, lymphoplasmacytic meningiomas stains positive for epithelial membrane antigen, and Langerhan’s histiocytosis stains positive for CD1a. Histiocytosis is distinguished by bony involvement and extensive eosinophilia. Lymphomas conversely show cellular atypia and occasionally erythrophagocytosis. 

Treatment of RDD is largely empiric due to its low incidence. Surgical extirpation of intra-cranial RDD is facilitated by a well defined tumor-parenchymal margin but should be balanced against risks based on location, especially for skull base lesions. Gross total resection offers curative goals, while subtotal resection risks recurrence and growth. Adjuvant or stand-alone radiation, chemotherapy, and steroids are considerations, but all are not well supported [1]. Lastly, therapeutic neglect in hopes of spontaneous remission is viable, but not advised in lieu of neurologic deficits or uncertain diagnosis.

The prognosis for RDD is overall good. The disease itself is benign and self-limiting; although patients may be symptomatic depending on location, or have undesirable cosmesis. Spontaneous resolution of non-CNS RDD has been reported [1]. Surveillance is up to the discretion of the treating physician and should be strongly considered in cases of subtotal resection or persistent CNS RDD.

## Conclusions

In conclusion, we present a rare case disseminated RDD with multiple extra-axial lesions. The differential for dural based masses dictates patient care and includes benign and malignant etiologies; and although uncommon, RDD should be considered. Maximum safe surgical resection offers definitive, rapid treatment of CNS RDD. This case report may be used in aggregate to further define the natural history and durable therapeutic approaches for RDD.
